# Evidence of sheeppox in seventeenth-to nineteenth-century France: a multi-disciplinary investigation of a sheep mass mortality assemblage

**DOI:** 10.1098/rsos.252320

**Published:** 2026-06-10

**Authors:** Annelise Binois-Roman, Louis L’Hôte, Roisin Ferguson, Kevin G. Daly

**Affiliations:** 1 Ecole d’Histoire de l’Art et d’Archéologie de la Sorbonne, Université Paris 1 Panthéon-Sorbonne, Paris, Île-de-France, France; 2 UMR 7041 Archéologie et Sciences de l’Antiquité, CNRS, Nanterre, Île-de-France, France; 3 UCD School of Agriculture and Food Science; 4 UCD Conway Institute of Biomolecular and Biomedical Research, University College Dublin, Dublin, Republic of Ireland; 5 School of Biological Sciences, University of East Anglia, Norwich, UK; 6 Smurfit Institute of Genetics, Trinity College Dublin, Dublin, Republic of Ireland

**Keywords:** palaeopathology, ancient DNA, zooarchaeology, veterinary history, sheep (*Ovis aries*), epizootic disease, ancient pathogens, sheeppox, *Taenia hydatigena*

## Abstract

Epizootic outbreaks posed major threats to food security, economic stability and animal welfare in past communities. While these events and their impacts are well documented in historical records, very few archaeological examples are known, and none for which the causal agent has been reliably identified. We therefore investigated a seventeenth-to nineteenth-century ovine mass-mortality assemblage from Louvres, France, using an interdisciplinary approach integrating palaeogenetics, zooarchaeology, history and veterinary science. The deposit contained nine complete, articulated sheep skeletons, mostly older individuals of both sexes, which were unbutchered but displayed flaying marks. Aside from minor age-related lesions and one ectopic bone nodule, the animals exhibited good skeletal health, with no obvious cause of death. Ancient DNA analysis was therefore conducted and proved highly successful, conclusively identifying two ovine pathogens: sheeppox virus (SPPV) and the parasite *Taenia hydatigena*. When considered alongside archaeological, veterinary and historical evidence, these findings indicate that the animals died during a sheeppox outbreak. This study provides, to our knowledge, the first detection of SPPV in archaeological bone and the first palaeogenome of this virus. It also offers new insights into livestock health, mortality, and disease management in early modern France, demonstrating the value of integrated approaches for reconstructing past epizootics.

## 1 Introduction

‘*Thicker than squalls swept by a hurricane from off the sea, plagues sweep through flocks; and not one by one diseases pick them off, but at a stroke, a summer’s fold, present and future hopes, the whole stock, root and branch.’* (Virgil, Georgics III, ll [1, pp. 471-473]). Written by Virgil in the first century BCE, these verses highlight well the major concern that epizootic outbreaks represented for ancient sheep farmers, and rightly so: many depended on the flock’s production—including wool, cheese and meat—for their subsistence, and the loss of their animals would have had devastating economic and sanitary consequences. Animal disease can still have similar impacts on societies today, an issue which the One Health framework developed at the turn of the millennium has brought into focus in recent years [2]. However, although the effect of animal disease on present-day societies—whether through direct zoonotic transmission or indirect economic and social repercussions—has been studied in depth, our understanding of its impact on past societies, who relied more heavily on animal resources and lacked our modern medical arsenal, remains fairly limited.

Textual evidence of epizootic animal mortalities is encountered in almost every written civilization and invariably highlights the severity of these events [3–5]. Material and skeletal evidence is, on the other hand, few and far between, its study plagued by technical and methodological issues. The disarticulated, butchered state in which animal remains are typically found is an important limiting factor, but even mass animal burials are often difficult to interpret, requiring a distinction between disease, ritual depositions and accidental mortalities [6,7]. Recent advances in palaeogenetics have facilitated the previously near-impossible task of identifying pathogenic organisms in animal remains, with analyses now sensitive enough to detect traces of pathogen genomes both bacterial and viral [8– 10]. However, demonstrating infection by an agent does not necessarily translate into the host having died from that specific cause, compounding the issue.

We nonetheless suggest that, given the right circumstances, archaeological epizootic mortalities can be reliably identified by an interdisciplinary approach relying on archaeological, osteological, palaeogenetic, historical and veterinary lines of evidence—a research strategy falling within the One Paleopathology framework [11,12]. This paper presents how this systematic approach was applied to a sheep mass burial from historical northern France and allowed the identification of the animals’ cause of death—specifically here, an outbreak of sheeppox. This unprecedented result offers new insights into livestock health, mortality and disease management in seventeenth- to nineteenth-century France, demonstrating the potential of integrated approaches in reconstructing past epizootics and enhancing our understanding of both past disease expression and societal responses.

## 2 Archaeological context

**Figure 1:**
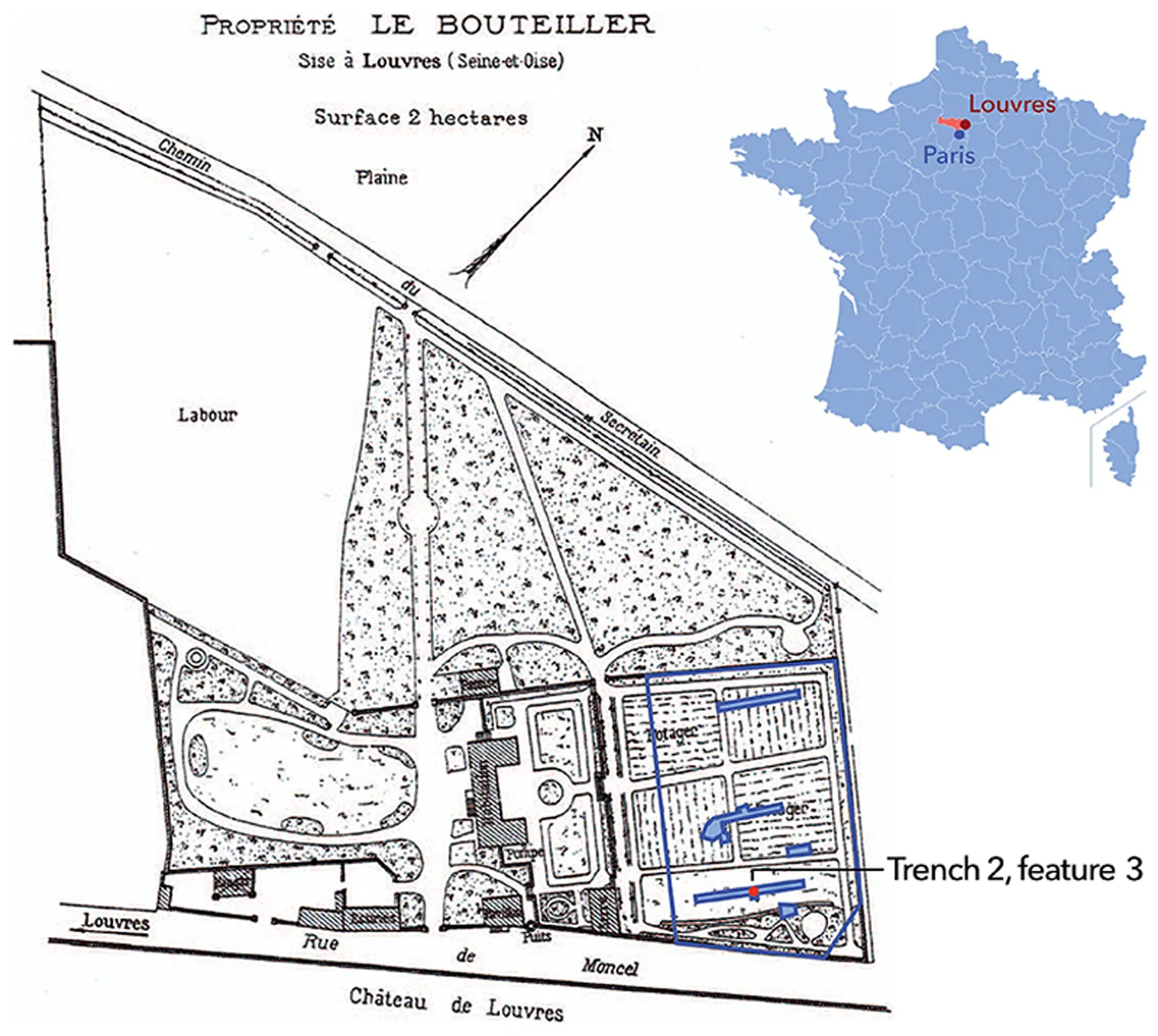
Nineteenth-century map of ‘le Bouteiller’ farm in Louvres, France (Bibliothèque du Musée Condé, Chantilly, in [13]). Location of the surveyed plot indicated by blue outline, the five trenches in light blue and feature 3 by a red dot.

**Figure 2:**
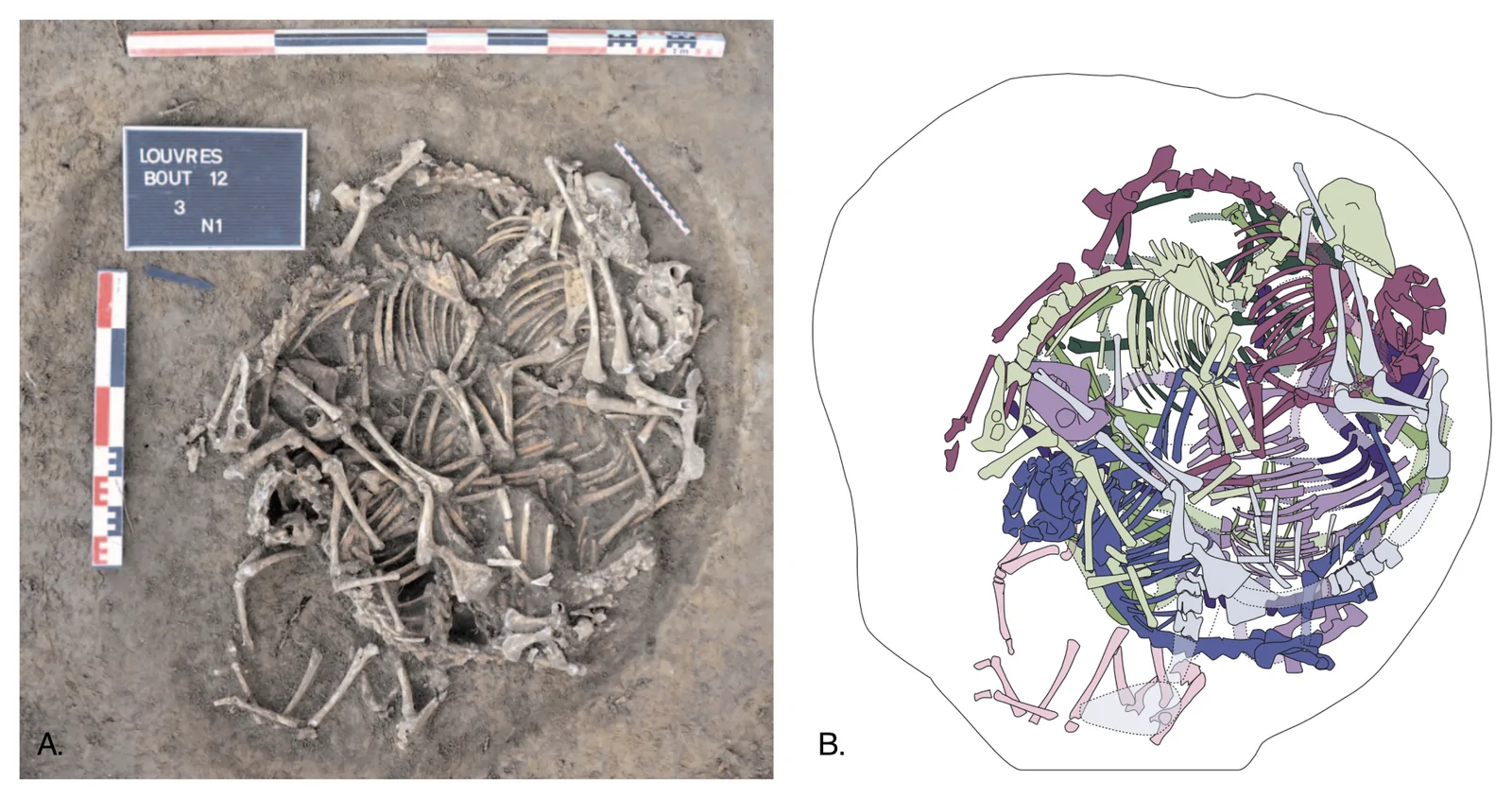
Feature 3, Louvres ‘le Bouteiller’ 2012 excavations. (A) Photograph of the excavated pit (courtesy of A. Konopka) and (B) illustration of the individual skeletons.

In 2012, a small-scale archaeological assessment was carried out by the INRAP (France’s National Institute for Preventive Archaeological Research) on the ‘le Bouteiller’ farm in Louvres, France, about 25 km north of Paris; we refer to the archaeological assemblage as Louvres ‘le Bouteiller’ from this point onwards. Five trenches were dug in what used to be the farm’s vegetable garden ([13]; figure 1). A bowl-shaped pit (feature 3, *ca* 1.1 m diameter × 0.4 m deep) was brought to light and found to contain articulated animal skeletons (figure 2). No other noteworthy features were identified by the survey, and no excavations of the plot were mandated.

The skeletons were radiocarbon dated to the seventeenth to twentieth century CE in a discontinuous probability distribution (175 ± 30 BP, Poz-85860, calibration 2σ IntCal20 [14] in the electronic supplementary material, table S1 and figure S1). Archival evidence of land use and occupation suggests the most recent end of the distribution to be unlikely, and a probable deposit date of 1658–1888 cal CE was retained [15].

Careful excavation and a thorough photographic documentation of the dismantlement of the bone accumulation allowed the identification of nine skeletons, which were numbered top-down in reverse deposition order. The animals lay on their sides with extended limbs and appeared to have been organized in the pit to optimize its infilling, with vertebral columns aligned on the edges and limbs oriented towards the centre in a circular pattern (figure 2). Excavation data suggest the carcasses were deposited in a single episode and that the pit, apparently purpose-dug for the event, was immediately filled in [15].

The bone accumulation was dismantled partly by individuals, partly collectively, and not all elements could be reattributed to specific skeletons post-excavation; individuals therefore comprise between 28 and 98 elements each, with over 250 elements (mostly vertebrae, ribs and phalanges) remaining non-attributed (table 1). Bones were often complete with intact surfaces, though the three lower-most skeletons presented some brittle elements with partly dissolved surfaces and low bone density.

As the skeletons showed relatively high completeness, a zooarchaeological examination (see §6) allowed all the animals to be identified as domestic sheep (*Ovis aries* L. 1758) of average size and slender constitution (average metacarpal slenderness index 10.3%; [15]). Their estimated withers height varies between 54.9 and 65.3 cm (table 1), consistent with recorded heights for northern France at the time (Louvres ‘le Bouteiller’ average: 60.4 cm; seventeenth to eighteenth century average: 59.0 cm; eighteenth to nineteenth century average: 63.5 cm; [16]). Sex was identified from osteological criteria on the pelvis, then confirmed by DNA for six individuals (see §6 and table 1); five females and four males were identified, all of whom were hornless. The male individuals displayed fairly subtle masculine characteristics for their age, and it is likely all were castrated, as was traditional in eighteenth- to nineteenth-century French farming systems [17]. Age at death indicated an overall mature population, consisting of five older adults (>7–8 years old), three adults (4.5–8 years old) and one subadult (2.5–3.5 years old) [15]. This demographic pattern, in which a high number of older males are retained in the flock, is suggestive of a farming system with a strong focus on the production of wool, as can be observed ethnographically in some traditional systems [18].

Cut marks were observed on two skeletons [15]. In sheep no. 5, both radii exhibited a deep incision on the proximal diaphysis, oriented perpendicular to the shaft. As the carcass was otherwise unbutchered and fully articulated, these marks are interpreted as traces of flaying, albeit situated unusually high on the limbs. In sheep no. 2, incisions were recorded on the atlas, consisting of a series of fine, parallel cuts on the underside of the atlanto-occipital joint. Given that the head and neck were found in close articulation, these traces cannot be attributed to beheading. Instead, they are most plausibly interpreted as evidence of slaughter by throat-slitting. However, such a procedure typically produces a single incision lower down the cervical column; the multiple cuts observed here indicate a repeated action unnecessary for the purpose. The incisions on sheep no. 2’s neck are therefore either atypical examples of slaughter marks or the result of an alternative activity that remains to be identified.

The sheep from the pit displayed good skeletal health for animals their age [15]. Four individuals showed no pathological modifications, and four showed only minor healed lesions (table 1). Only sheep no. 2 presented any pathological element of note, a small calcified nodule that was found stored with its head and cervical column; its skeleton was otherwise healthy.

## 3 Study aims and materials

The sheep burial of Louvres ‘le Bouteiller’ immediately struck archaeologists as atypical. Indeed, burial is almost never the intended outcome of a farm animal’s life; whether after a brief fattening period or a lifetime of labour, most are ultimately slaughtered, butchered and consumed. Multiple burials—in which several individuals are interred together in a single event—are even rarer, and almost never occur outside of two broad phenomena: mortality crises and ritual depositions [15]. Our study aimed, therefore, to identify the nature of the Louvres ‘le Bouteiller’ sheep deposit, and if mortality-related, to explore the animals’ cause of death.

The first question was fairly simple to address. As outlined in previous work by Binois-Roman [15], animal burials need to meet the following criteria to qualify as mass mortality events: victims belonging to a single species; in numbers such that unrelated, concurrent deaths are highly improbable; the deaths of which can be demonstrated to have been simultaneous; and whose burial shows no evidence of ritual activity. The Louvres ‘le Bouteiller’ deposit meets all of these criteria, with nine sheep found densely packed within a single feature. The high degree of carcass entanglement, the perfect anatomical articulation even in the deepest layers, and the absence of weathering or scavenging marks indicate a single act of deposition followed by immediate backfilling. No material evidence of ritual activity was observed; modern-period Catholic France is moreover an unlikely setting for such activities. We therefore concluded that the Louvres ‘le Bouteiller’ sheep represented the victims of a mass mortality event.

In consequence, this paper focuses on identifying the origin of said mortality event, as sheep are susceptible to a variety of causes of death. While epizootic disease is, of course, common, historical evidence suggests that illnesses only accounted for about half of mass deaths in pre-industrial times, with as many events arising from harsh winters, springtime storms, floods, intoxications and predator attacks [15]. Most of these occurrences do not, however, leave characteristic skeletal lesions on the bones—if any at all. Our diagnostic approach needed, therefore, to address a very wide scope of causes of death in the absence of specific palaeopathological evidence.

Two main lines of enquiry were adopted. On one side, written records from modern-period northern France were examined to identify the most frequent types of sheep mortality and their clinical manifestations in that specific context. A palaeo-epidemiological analysis of the skeletal assemblage was then carried out to identify commonalities and inconsistencies with the collected data, in order to suggest possible diagnostic hypotheses (see §6). In parallel, palaeogenomic analyses were undertaken to search for biomolecular traces of pathogens in selected samples from the sheep assemblage. The latter were chosen as to maximize chances of detecting pathogen DNA; we opted to sample a petrous bone from all available crania, teeth presenting intact closed roots from six individuals, as well as a dental calculus sample from one individual and the pathological ossified nodule associated with sheep no. 2, for a total of 16 analysed samples (table 2; electronic supplementary material, table S3). Finally, results from all lines of evidence were integrated to support a final diagnosis of the sheep’s cause of death.

## 4 Results

### 4.1 Analysis of host DNA

Although the focus of the palaeogenomic analysis was to identify the presence of potential pathogen genetic material, the methods employed for DNA analysis also allowed access to information regarding the sheep hosts themselves. All samples were run through an initial shotgun sequencing analysis following the protocol outlined in §6; each yielded sufficient amounts of endogenous DNA for species confirmation and postmortem damage patterns at read ends supporting the authenticity of the host ancient DNA (table 2; electronic supplementary material, figure S3).

The results confirmed that of the osteological analysis, all endogenous content of the samples mapped to sheep (*O. aries*), as expected, and sex, when available, was consistent with osteological sex, confirming the robustness of osteological methods when dealing with complete skeletons. The host genome of the pathological nodule was also confirmed to be sheep, with an endogenous DNA content of 22.36%, consistent with previous reports for nodule remains [19]. It stemmed from a female individual and shared a mitochondrial DNA (mtDNA) haplotype with sheep no. 2 (electronic supplementary material, figure S2).

As several female-line relatives were potentially present in the assemblage, and as the nodule was not attached to any skeletal element, we wished to confirm genetically its attribution to sheep no. 2. A *D*-statistic test [20] using ANGSD [21] was performed between sheep-aligned DNA from the nodule and four of the Louvres ‘le Bouteiller’ sheep (electronic supplementary material, table S4), to test for greater derived allele sharing of an H3 genome with either of an H1 or H2 genome. We posited that in tests where the nodule and the animal host of the node were included, there would be a substantial excess of ‘derived’ genetic variants shared between these two samples. The test yielded an average absolute *D*-statistic of 0.588 when the nodule (RF001) was placed in the H3 position and sheep no. 2 (RF003) was placed in the H2 position, while tests without sheep no. 2 ranged in absolute *D*-values of 0.0005–0.0294. This strongly suggests that the two samples (RF001 and RF003) are genetically identical and allow secure attribution of the nodule to sheep no. 2.

### 4.2 Analysis of the pathological nodule

As ossified nodules are rare in the archaeozoological record, the specimen was examined as the first potential candidate for the etiological agent of this mass mortality event and assessed using a range of analytical approaches.

**Figure 3:**
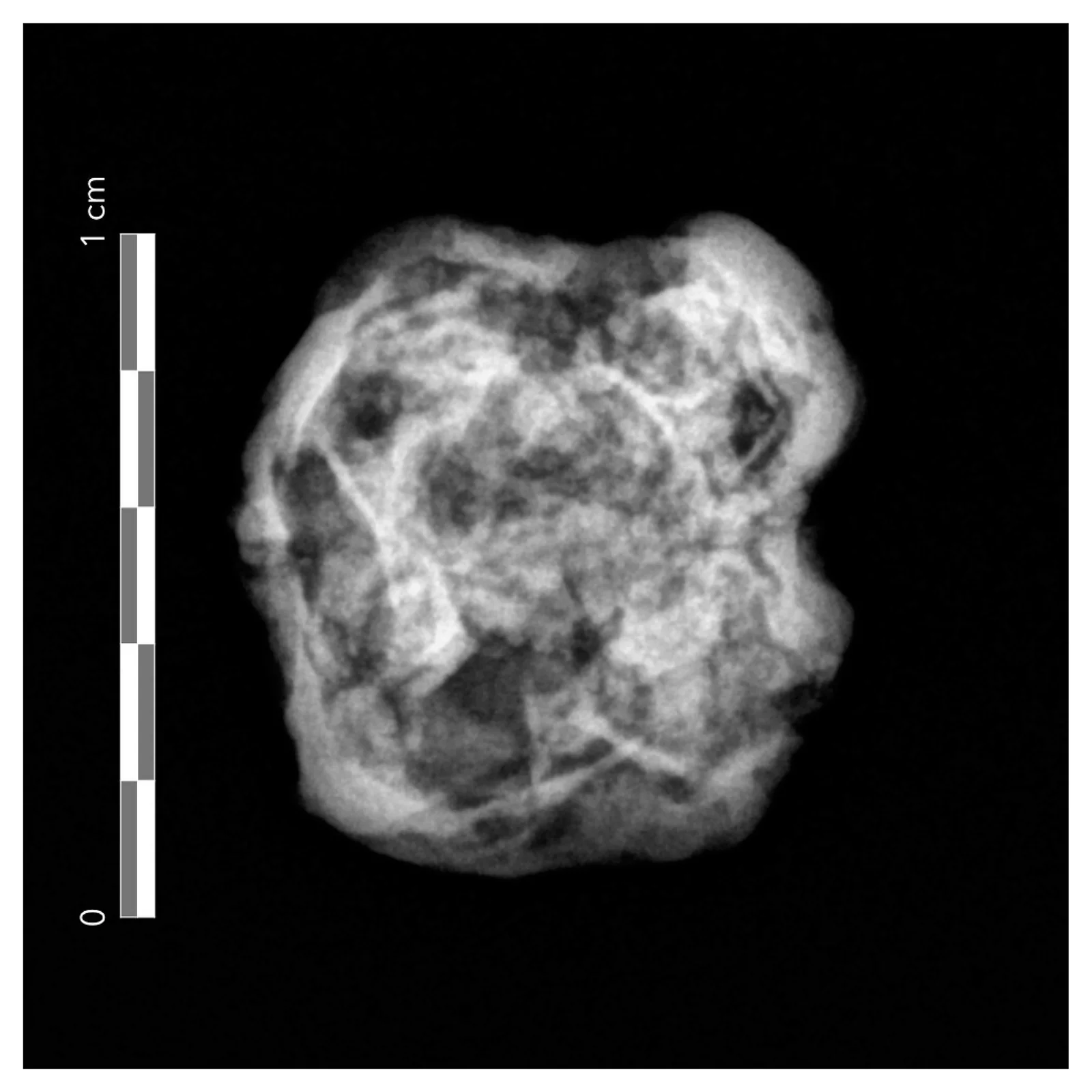
Radiograph of the nodule found with individual 2, Louvres ‘le Bouteiller’. Approximate scale overlaid onto radiograph.

The nodule was recovered from a bag of cranial debris from sheep no. 2 and was initially presumed to have originated from the cranial or cervical region of that animal (see the electronic supplementary material, Information note 1). It was a flattened, sub-circular biological calcification measuring approximately 1 cm in diameter and 0.4 cm in height. Its surface was beige and bone-like, smooth in texture but uneven in form and punctured by several sub-circular perforations with rounded edges. The specimen was of very low density and appeared at least partially hollow. Although no photograph of the object is available, its external appearance closely resembled that of the biological artefact illustrated by Komar & Buikstra [22]. Radiographs taken using a dental radiographic machine revealed a heterogeneous, poorly organized structure composed of voids and snowy areas traversed by a few denser lines (figure 3). The radiographic density of the material matched that of bone.

The nodule’s appearance and presumed anatomical origin suggested a primary diagnostic hypothesis of a calcified cervical lymph node, which is most often in sheep caused by caseous lymphadenitis, an infectious disease owing to *Corynebacterium pseudotuberculosis* (see the electronic supplementary material, Information note 1). A palaeogenetic investigation of the specimen was therefore undertaken to explore this diagnostic hypothesis (see §6).

**Figure 4:**
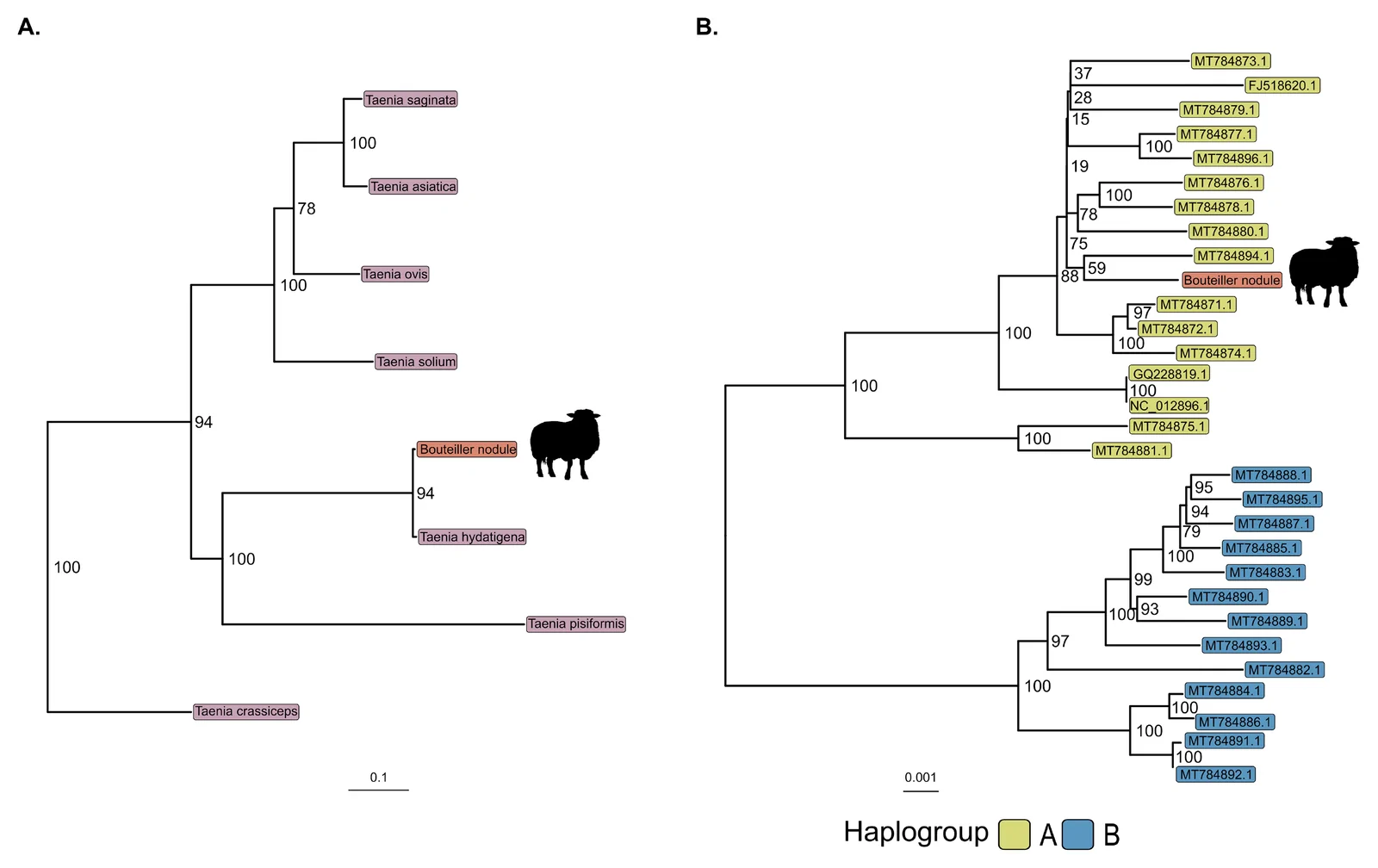
Phylogenetic placement of the Louvres ‘le Bouteiller’ nodule isolate among *Taenia* species and mtDNA haplogroups. (A) Phylogenetic placement of the Louvres ‘le Bouteiller’ taenid mitochondrial genome within the diversity of *Taenia* species. A maximum-likelihood phylogeny was constructed with IQ-T_REE_2 [24] using complete mitochondrial genomes from representative *Taenia* species. Bootstrap support values are shown at the corresponding nodes. The nodule sequence is highlighted in red and accompanied by a sheep silhouette. (B) Haplogroup assignment of the Louvres ‘le Bouteiller’ taenid mitochondrion. A high-resolution maximum likelihood mitochondrial haplogroup tree of *T. hydatigena* was generated with IQ-T_REE_2. Samples are coloured according to haplogroup (A = yellow, B = blue), and tip labels correspond to GenBank accession numbers (electronic supplementary material, table S7). The nodule sequence (red) clusters within haplogroup A. Bootstrap support values are shown at the corresponding nodes.

The host genome was aligned to sheep and confirmed to be of ancient origin through damage pattern mapping (electronic supplementary material, figure S3). Unaligned reads were examined using a unique *k*-mer counting approach [23]. Surprisingly, whilst no DNA from infectious bacteria was identified, abundant *k*-mers were assigned to the parasitic tapeworm *Taenia hydatigena* (*E*-value = 1.48). After alignment to the *T. hydatigena* reference genome (Thy_genome_v2.1_HiC), reads showed typical postmortem deamination and a descending edit distance indicative of a close match between DNA and reference genome (electronic supplementary material, figure S3). The resulting alignment represented 0.03× genome coverage (182 013 reads mapped) and 48.26× mitochondrial coverage (electronic supplementary material, figures S4 and S5). After further sequencing with Uracil DNA-glycosylase (UDG)-treated libraries to remove post-mortem damage, we produced a 0.24**×**-fold coverage nuclear genome and a mitochondrial genome of 130.8**×**-fold (electronic supplementary material, figures S6 and S7), enabling phylogenetic confirmation of the species as *T. hydatigena* and placement within the A haplogroup (figure 4B).

*Taenia hydatigena* is today in Europe an uncommon intestinal parasite of domestic dogs and other carnivores, having largely disappeared from industrialized countries in the twentieth century owing to antiparasitic treatments and changes in animal-feeding practices. The parasite is acquired by the carnivorous final host through the consumption of the raw viscera of large herbivores, who serve as intermediate hosts for this parasite. As such, sheep and goats, and less commonly cattle, deer and pigs, will host the larval stage of the flatworm, previously known as *Cysticercus tenuicollis*, on the peritoneal surface of their abdominal organs, most notably the liver, the spleen, the mesentery and the omentum [25–27]. The larvae form a bladder-like cyst of 1–7 cm diameter, attached to the organs by a thin stem; in sheep, the cysts can also more rarely develop inside the liver. The disease in ruminants is chronic, and the cysts may calcify over time [28,29].

No direct prevalence data is available for *T. hydatigena* in seventeenth-to nineteenth-century France, but the parasite was probably widespread. It remains common where wild carnivores and stray dogs act as reservoirs and livestock are not routinely treated; reported prevalence in sheep ranges from 2% (India [30]) to over 50% (Tanzania [31], Ethiopia [32]), and in dogs from around 10% (Vietnam [33], Italy [34]) to over 60% (Mongolia, Dazan 1978 in Temuujin 2022 [35]). Genomic evidence of the parasite has also been found in archaeological contexts, with identifications reported from a Danish Iron Age pond [36], from medieval Norse Greenland [37] and from early Modern urban refuse layers in Denmark and Lithuania [38]. As taeniid eggs cannot be distinguished to species level morphologically, no specific palaeoparasitological evidence of *T. hydatigena* eggs is available, but they are probably included among the many taeniid eggs identified in archaeological deposits worldwide.

The identification of the nodule as a calcified parasitic cyst of a *T. hydatigena* larva is therefore consistent with most of our data. This parasite is a documented source of calcified cysts in sheep and appears to have been common at the time and place of the Louvres burial. No literature regarding calcified *T. hydatigena* cyst morphology was located, probably owing to its limited diagnostic relevance, but published descriptions of other taeniid calcified cysts, notably of *Echinococcus* sp., match the Louvres specimen reasonably well. The only inconsistent diagnostic element was the presumed anatomical location. While aberrant cyst localizations have been recorded in sheep [39,40], none are in the head or cervical region, which are physiologically unsuitable for larval development. It is therefore likely that the specimen was misassigned during excavation or post-excavation handling.

To our knowledge, this is the first archaeological identification of a *T. hydatigena* cyst and the first confirmed animal parasitic cyst in the archaeological record. The species is mentioned by Baker & Brothwell as a potential source of archaeological pathological specimens, but these are considered ‘most unlikely to be recovered’ [41, p.172]. Parasitic cysts have been reported in human remains—mostly interpreted as *Echinococcus* sp. infestations—at Neolithic Siberian [42], medieval Italian [43], and medieval Spanish sites [44,45], though these specimens tend to be larger and hollower than the Louvres ‘le Bouteiller’ example.

In sheep, *T. hydatigena* infestation is generally asymptomatic and usually detected incidentally at slaughter. Adult sheep may experience mild production losses, and severe infestations can occasionally cause sporadic deaths in lambs, but such events are rare [34,46]. Even so, the complaint is an individual one, never resulting in multiple deaths, especially not in mature adults. It therefore seems highly unlikely that the infestation documented in Louvres ‘le Bouteiller’ could have resulted in the death of sheep no. 2, let alone nine adult sheep over a short span of time. It is even more implausible that the sheep would have been culled and discarded because of a parasitic infestation. Before the late-nine-teenth-century understanding of helminth lifecycles and the mid-twentieth-century introduction of synthetic antiparasitic drugs, parasitic burdens in European livestock were extremely high. Most, if not all, animals would have harboured multiple worm species without raising concern, and infestations were rarely noted prior to slaughter. The Louvres ‘le Bouteiller’ *T. hydatigena* cyst is therefore best interpreted as an incidental finding of intercurrent disease, unrelated to the primary cause of death.

### 4.3 Analysis of the skeletal assemblage

If the nodule could not provide answers to the cause of death, perhaps the skeletons would. We first approached the diagnosis through a palaeo-epidemiological angle: having identified in relevant historical sources the most common causes of sheep mass mortalities in the Modern period, we proceeded to map the epidemiological characteristics of each, including terrain, seasonality and demography of victims (see §6). The resulting profiles were then compared to that of the Louvres ‘le Bouteiller’ deposit in order to identify the diagnostic hypotheses that most closely matched our observations.

Unfortunately, our differential diagnosis did not lead to any conclusive results. The lack of data in Louvres ‘le Bouteiller’ relating to the season of death hindered our approach, and the atypical demographic profile, highly skewed towards older animals, contradicted most diagnostic hypotheses. Apart from the average age at death, the epidemiological profile of the deposit was fairly unspecific (electronic supplementary material, table S2); agreement was weakest with pathological causes (anthrax, liver fluke, sheeppox, red disease) and a toxic or accidental incident was initially tentatively suggested [15].

These unsatisfactory results were therefore complemented with a palaeogenomic approach. As previously, unaligned reads were then examined for pathogen DNA sequences using *k*-mer matching [23]; whereas the petrous samples yielded no positive results, four teeth samples yielded varying amounts of reads for the sheeppox virus (SPPV; *Poxviridae*): 440 reads (sheep no. 2), 302 reads (sheep no. 1), 69 reads (sheep no. 9) and 12 reads (sheep no. 4) (table 3). Alignment-based assessment of this screening data [47] suggested the unambiguous presence of SPPV reads (electronic supplementary material, figures S11). The absence of viral reads in petrous samples probably reflects tissue-specific pathogen distribution and differences in DNA preservation; teeth, particularly dental pulp and surrounding tissues, are vascularized and may capture blood-borne pathogens during systemic infection, whereas the petrous bone is less likely to preserve pathogen DNA despite its excellent preservation of host DNA. We additionally observed seven reads matching SPPV in dental calculus from sheep no. 2 (electronic supplementary material, table S3), suggesting it is possible to detect this pathogen in calcified plaque from infected individuals.

Among the four SPPV-positive samples identified during the initial screening, sheep no. 2 yielded the highest number of reads aligning to SPPV and was therefore selected for targeted genome enrichment (see §6), resulting in high genome coverage (mean depth 263.01×) with complete genome recovery (100% breadth at ≥1×; table 3). The SPPV-positive teeth from sheep no. 1 and sheep no. 9 were subsequently re-sequenced following UDG treatment to reduce postmortem-associated damage, yielding high genome coverage for sheep no. 1 (mean depth 12.10×, 99.9% breadth at ≥1×) and lower but still informative coverage for sheep no. 9 (mean depth 1.55×, 77.2% breadth at ≥1×; table 3). Genome-wide coverage profiles indicate that reads are distributed across the viral genome rather than concentrated in specific regions (electronic supplementary material, figure S12). A coverage peak is observed around the inverted terminal repeat (ITR) region, as one of the duplicated ITR copies was removed from the reference genome to allow read mapping across this region (see §6; electronic supplementary material, figure S12). Mapping statistics, including edit distance distributions, nucleotide misincorporation patterns and read length distributions, further support the authenticity of the recovered viral sequences (electronic supplementary material, figure S12).

The recovery of SPPV genomes from these three individuals, spanning depths from 1.55× to 263.01× and covering substantial portions of the viral genome, strongly suggests that these animals were infected with sheeppox peri-mortem. Sheep no. 4 yielded too few viral reads in the initial shotgun screening to enable efficient genome reconstruction following deep sequencing and was therefore not pursued further. While sheep no. 4 may also have been experiencing an active SPPV infection at the time of death, the limited number of viral reads prevented reliable genome reconstruction in this case.

**Figure 5:**
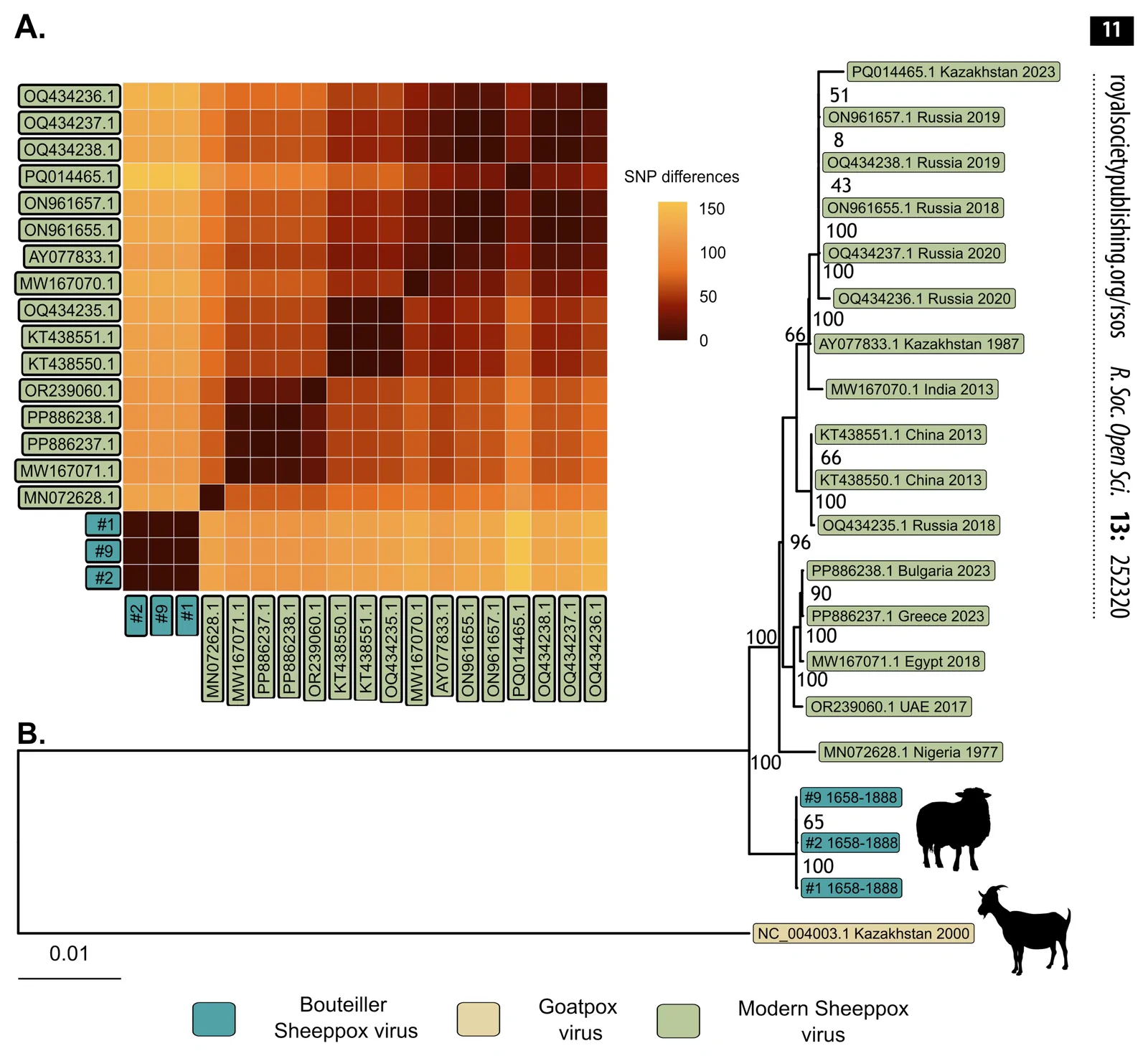
Genomic relatedness and phylogenetic placement of the Bouteiller sheeppox virus (SPPV) isolates. (A) Pairwise SNP distance matrix among SPPV genomes. Colours indicate the number of SNP differences between each pair of sequences, with darker shades representing higher divergence, showing genomic relatedness between the Louvres ‘le Bouteiller’ SPPV isolates (nos. 1, 2 and 9). Modern isolates are represented by their corresponding GenBank accession numbers matching the isolates from (B). (B) Phylogenetic position of the Louvres ‘le Bouteiller’ SPPV isolates relative to modern diversity. An SNP-based maximum likelihood phylogeny [24] based on complete genomes, rooted on goatpox virus (GTPV). Tip labels show accession numbers and sampling locations and dates. Louvres ‘le Bouteiller’ genomes are represented in blue with a sheep silhouette, and the modern SPPV lineage in green. The outgroup GTPV is represented in beige and with a goat silhouette. Bootstrap values (from 1000 ultrafast replicates) are displayed at their respective nodes.

To assess the relatedness between the recovered Louvres ‘le Bouteiller’ SPPV genomes and SPPV today, a pairwise distance matrix was computed using single-nucleotide polymorphisms (SNPs), representing positions differing between genomes (figure 5A). Only two SNPs were observed among the isolates: one unique to sheep no. 1, and one unique to sheep no. 2; when we directly examined these loci, both appeared to be false positive calls (see the electronic supplementary material, figures S13 and S14, and note 2), and our recovered SPPV genomes are probably identical. In comparison, 122 SNPs are observed on average between the Louvres ‘le Bouteiller’ genomes and modern SPPV, and on average 44 SNPs differentiate pairs of modern SPPV genomes. The recovery of highly similar viral genomes from multiple individuals at the Louvres ‘le Bouteiller’ site suggests that the sheep were infected during a single outbreak. Construction of a maximum likelihood phylogenetic tree [24] with 16 modern SPPV sequences and one goatpox virus (GTPV) as an outgroup shows that Louvres ‘le Bouteiller’ SPPV strains form a distinct, basal monophyletic clade to modern SPPV lineage, consistent with a placement of sequences from the seventeenth to nineteenth century CE (figure 5B), as opposed to modern contaminant SPPV genomes.

## 5 Discussion

### 5.1 Sheeppox: epidemiology and clinical presentation

The SPPV is a double-stranded DNA virus of the *Capripoxvirus* genus, within the *Poxviridae* family. It is closely related to GTPV and lumpy skin disease virus (LSDV), and more loosely to other members of *Poxviridae*, such as smallpox (variola) or cowpox (vaccinia). Its genome of *ca* 150 kbp [48], large for a virus, makes it a good candidate for detection in archaeological material. No report of SPPV identification in archaeological bone has, however, of yet been published (although *Capripoxvirus* may have been detected from historical manuscripts, see [49]), and this is to the best of our knowledge the first report of a palaeogenome of the virus.

Sheeppox is the disease determined in sheep by the SPPV virus. Though other animal species may host the virus, the disease itself is in natural conditions only observed in sheep and very occasionally in goats; cattle appear impervious to most strains [50]. Sheeppox is extremely contagious among sheep, and morbidity in a naïve flock is usually 75–100% [51]. It is transmitted through direct contact and infectious aerosols (nasal discharge, saliva, scabs); contagion often occurs during gatherings of animals on communal pastures or at watering points, at fairs and markets, or with the introduction of a new individual in a flock. Mortality is high but variable, with average death rates ranging from 5 to 85% of affected individuals [51,52].

Two main forms of the disease are distinguished. In the regular or benign form, more common in adults, animals initially exhibit fever, loss of appetite and ocular discharge, followed after four to five days by the eruption of cutaneous papules on the areas of the body not covered in wool, most notably the face. These papules develop into vesicles which subsequently rupture, spreading infectious fluid and are replaced by nail-like scabs that eventually fall off, leaving permanent scars [53]. Most animals survive, and mortality rates rarely exceed 5% [51]. The malignant form is most frequently observed in animals under two years of age, which represents the most common form. The invasion phase is more severe, with marked depression and prostration, and some lambs may die even before the appearance of the papules. The latter affects not only the woolless areas of the skin, but also the mucous membranes and internal organs, and spreads to the mouth, lungs, trachea, digestive and urogenital mucosae. Animals may exhibit respiratory difficulties, pneumonia, and haemorrhagic diarrhoea, and pregnant females almost invariably abort. In some cases, the eruption may involve the entire skin surface. Mortality rates from malignant sheeppox range from 50 to 85% of affected animals [51–53]. In survivors of both forms, sheeppox confers a strong and lasting immunity that prevents reinfection.

Terrain has little influence over the prevalence of sheeppox, which mostly depends on local sheep density and the intensity of livestock movement and trade. Its seasonality is debated; the disease seems possible all year round, but various authors point to different preferred seasons, perhaps owing to local herding practices and commercial circumstances. Certain breeds are more resistant than others; wool sheep show greater susceptibility than hair sheep, and breeds specifically selected for fine, dense wool such as the merinos are particularly vulnerable. Age is nonetheless the greatest risk factor, with deaths highest in animals aged under 2 years, and more so between 4 and 12–18 months of age: mortality rates can reach 80% in that cohort while most adults of the same flock survive [53]. Among adults, health status is the main risk factor, with aged individuals, those weakened by intercurrent disease, and ewes in late pregnancy displaying higher mortality rates [54]. Females appear slightly more at risk than males [53,54].

As sheeppox does not affect the skeletal system, the disease leaves no identifiable traces on archaeological bones. The skin lesions, however, severely devalue the hides, sometimes rendering them unusable [51], and the repulsive appearance of the lesions may discourage the consumption of the carcasses.

### 5.2 The history of sheeppox in France

Owing to its characteristic dermatological symptoms and highly contagious nature, sheeppox is easily identified both in live animals and in written accounts and cannot be mistaken in its typical presentation. Ancient textual evidence can therefore be considered fairly reliable. The earliest written description of the disease dates back to Roman antiquity, with Columella’s description of the disease of sheep known as ‘the pustule’ (*pusula*), an extremely contagious and highly lethal dermatological ovine disease (first century CE [55]). Sheeppox appears to have been fairly common in medieval western Europe. The disease appears in husbandry treatises such as Walter of Henley (thirteenth century England, [56]) and Jean de Brie (fourteenth century France, [57]), and is recorded as an explanation for sheep losses in accounting documents all through the thirteenth to fifteenth centuries in France and England [15]. The contagiousness and lethality of sheeppox were well recognized at the time, and as early as 1499, an ordinance of the *Mesta general de Castilla y León* (Spain) required shepherds to declare all places in which the disease had been observed [58].

In France, mentions remain common throughout the fifteenth and sixteenth centuries but appear to dip temporarily in the seventeenth century, possibly affording flocks a short respite [15]. The disease reappears, however, with a vengeance in the mid-eighteenth century, with epizootic outbreaks increasingly severe in both victim count and geographical spread attracting growing attention from the developing field of veterinary science. At least three treatises are specifically devoted to the disease during that period, as well as numerous chapters and papers [58–62]. In the same period, sheeppox also became the subject of public health policies, with rulings and decrees from 1714, 1778 and 1784 making it a notifiable disease and regulating sheep movement in areas of infection [63]. By the end of the eighteenth century, the disease had become so common that Claude Bourgelat, founder of the first veterinary school in 1761, ‘claimed that no animal ever reached the end of its life without having experienced sheep pox.’ [64, p. 454 (translated by A. Binois-Roman)].

In 1801, an ordinance from the Paris Police Prefecture was passed in response to an outbreak affecting the western and northern parts of the region, including Saint-Denis, a major livestock trade centre less than 20 km from Louvres [63]. In addition to organizing the isolation of the sick and prohibiting their sale, the text also prescribes how the victims’ carcasses should be managed: they ‘shall be buried on the same day with their skin and wool, at a depth of 1.34 meters (4 feet), outside the boundaries of the municipalities, all at the owners’ expense’ [63, p. 558-559 (translated by A. Binois-Roman)]. Steep fines awaited offenders.

Following the success of smallpox inoculation, experiments with sheeppox inoculation were attempted in the eighteenth century [65]; though mostly effective, the method never really caught on beyond scientific circles. In the late nineteenth century, the development of an effective and reliable vaccine was met with public success and, combined with stringent public health measures, enabled the eradication of sheeppox from mainland France at the beginning of the twentieth century [58].

### 5.3 Interpretation and discussion

In light of this information, the diagnosis of sheeppox obtained by palaeogenomic analysis appears consistent with the archaeological data from the Louvres ‘le Bouteiller’ assemblage. The disease is both highly contagious and deadly in sheep and could easily lead to the simultaneous deaths of nine individuals. Although SPPV infection was only confirmed in three animals, their situation within the pit (the two top-most and the bottom-most individuals), the archaeological evidence of a single burial event followed by quick infilling of the pit, the genomic evidence of a single viral strain, and the extreme contagiousness of sheeppox in naïve animals together point to all nine animals having been affected by the disease. The lack of evidence from the three other animals that were sampled is probably owing to differential preservation of the genetic material rather than absence of infection—it is indeed noteworthy that the viral material was only identified in one of the two teeth sampled from individual 1, and in no petrous bones including those from animals which yield SPPV-positive teeth (table 2).

The deposit also stems from an area and a period in which sheeppox was common: in addition to the 1801 outbreak referenced above, Delafond notes outbreaks in the Parisian area in 1773–1774, 1776, 1805 and 1810 [63]. Hurtrel d’Arboval further states that ‘In most of the departments of France, [the disease] returns epizootically only once in ten or fifteen years; but its recurrence is more frequent in the vicinity of Paris, where there is much passage, and where a considerable trade in sheep is carried on’ [64, p. 452 (translated by A. Binois-Roman)]. This reported frequency prevents us from ascribing the Louvres ‘le Bouteiller’ mortality to a specific epizootic event.

If place and time are therefore very typical, the same cannot be said for the demography of the victims. It is established that juveniles are most susceptible to sheeppox, and the majority of deaths are usually found within the 4–18 months cohort [53]. However, the youngest animal from Louvres ‘le Bouteiller’ was a subadult around three years old, and the remaining eight animals are mature adults, several of whom appear in their senior years. The population is therefore much older than expected, which explains the bad fit of the palaeo-epidemiological approach. No definitive explanation can be given for this discrepancy, but several factors might have played a part. It is possible that the farmer kept his lambs and yearlings on another site; it is also possible that he did not own any and only had a small finishing activity, acquiring cheap older animals and fattening them up for sale in the Paris market. The composition of the death assemblage is similarly uncertain: only 130 m^2^ of the farmyard were excavated during the assessment, and nothing precludes there being one or several other sheep-pits on the site, which may have included youngsters. This is unfortunately unverifiable, as the site now hosts a large residential building. Finally, it is conceivable that the Louvres ‘le Bouteiller’ sheep did not die from sheeppox but were culled out of the flock because of it—this would be in agreement with the slaughter marks observed on sheep no. 2. In that case, the owner could perhaps have decided to selectively cull the older infected animals but to try to save the younger ones.

After all, these sheep do fit the typical profile for adult victims of sheeppox. The latter are indeed most often individuals weakened by age or disease, which is very much in agreement with our results: at least four of the nine victims are identified as older adults above 6 years of age, and one, sheep no. 2, displayed strong evidence of *T. hydatigena* parasitism. High parasitic burdens are known to contribute to sheeppox mortality [53,59]. Though no information relating to *T. hydatigena* specifically is available, Hurtrel d’Arboval emphasizes the severity of the disease in cases of prior liver fluke (*Fasciola hepatica*) infestations [59], the symptoms of which *T. hydatigena* is reported to occasionally mimic [51]. Finally, the high proportion of adult males and the slender morphology of all animals suggest the flock might have been specialized towards the production of wool. Sheeppox is described as being more severe in breeds selected for fine wool, including in the merinos, which were popular around Paris at the time; though no evidence of the specific breed affinities of the Louvres ‘le Bouteiller’ sheep is available, this may have been a contributing factor.

While several hypotheses may account for the discrepancies in age profiles, a temporal evolution in disease presentation can be ruled out. The deaths occurred indeed recently enough that our analysis of sheeppox could rely on multiple, contemporaneous descriptions of its clinical manifestations [17,52,59,60,64].

The case also provides an opportunity to analyse how Modern period societies dealt with contagious disease among their livestock. The earliest legislation relative to sheeppox is dated in France to 1714, with several updates through the late eighteenth to early nineteenth century, which means that unless the deposit is dated from the earlier end of the probability distribution (1658–1698 CE (19.0%)), the farmer was probably aware of the existence of regulations—Louvres is not an isolated town. These regulations are possibly what led to the burial of the animals, though it was done only in partial compliance with local ordinances. Whereas the latter prescribe deep burials (4–6 French feet, i.e. 1.34–2 m) in areas outside the town, the Louvres ‘le Bouteiller’ sheep were interred directly within the farmyard, less than 60 m from the main house, in a pit that was originally only 60–80 cm deep. At least one animal had been flayed, in violation of all regulations—diseased skins and their trade being a known cause for the spread of the contagion. The diagnosis of sheeppox does allow us to better understand the atypicality of the flay marks on sheep no. 5: while they appear needlessly high up the limb for regular skinning marks, their location would have allowed cutting off the skin well into the woolled area, avoiding the pocks that would be found on the lower limbs, and therefore avoiding detection as a pocked skin. It is possible that several other individuals from the pit, perhaps all, were similarly flayed, but that a skilled skinner left no marks doing so. All in all, it seems as though the burial reflects only minimal compliance to regulations, either to provide an appearance of compliance, to save time, and to limit losses by selling skins, or out of ignorance of the regulations, perhaps because the burial predated their introduction. Finally, mention should be made of the possibly culled animal. If the traces that are observed are indeed slaughter marks, they could either stem from a mercy killing, intended to shorten the suffering of a dying animal, or from a sanitary precaution, eliminating a diseased sheep that could continue spreading the infection within the flock. Either act is possible within the cultural context, and both are documented in contemporaneous veterinary writings.

## 6 Conclusion

By combining archaeological, zooarchaeological, palaeopathological and genetic approaches, we disentangled the health status and likely cause of death of an ovine assemblage from Louvres, France, making it the first ancient animal mass mortality event to be genetically diagnosed. The responsible aetiological agent was identified as SPPV, cause of the severe viral disease sheeppox widespread among sheep in the region at the time. This study presents, to our knowledge, the first detection of SPPV in archaeological bone and the first palaeogenome of this virus, as well as the first genetic and palaeopathological evidence of *T. hydatigena* parasitism in sheep. These findings were made possible solely through an integrative, interdisciplinary approach, and our study therefore underscores the importance of cross-disciplinary collaboration in advancing our understanding of animal disease and welfare in the past.

The analytical framework we propose for future investigations aligns with the broader, recently developed concept of One Paleopathology [11,12,66]. This paradigm applies palaeopathological evidence—derived from human and animal skeletal remains, ancient DNA, and archaeological records—to illuminate the deep-time dynamics of health across humans, animals and ecosystems, with the explicit goal of informing modern public health policy, disease prevention and climate resilience strategies. Although the disease documented at Louvres ‘le Bouteiller’ is not zoonotic, it carried severe economic consequences for European societies until vaccination campaigns and public health measures led to its eradication in the twentieth century. The risk of resurgence, however, remains real, as recently illustrated in France by an epizootic outbreak of LSDV, its lesser-known, less severe sister disease [67,68]. Such a resurgence could have far-reaching economic, sanitary and social consequences, and a deeper understanding of SPPVs past evolution and historical dynamics may prove invaluable in shaping and informing current prevention and response strategies.

## 7 Methods

### 7.1 Zooarchaeological methods

The assemblage was analysed according to guidelines for best practices in zooarchaeology [69]; all bones were identified to anatomical part, weighed to a 0.1 g precision and measured with digital callipers following the recommendations of Von den Driesch [70]. Species distinction between sheep and goat was carried out using published criteria from [71,72], and sex identification was based on pelvic conformation relying on a combination of criteria from [73–76]. Wither height was assessed by averaging height reconstructions for all long bones from a given individual [77] and slenderness was calculated on the metacarpal using [78]. The sheep’s age-at-death was estimated through tooth eruption and wear for the animals who had preserved teeth [79] and relying on epiphyseal fusion stages for the remaining three individuals [3,5,7,80].

### 7.2 Palaeo-epidemiological methods

In previous work [15], evidence of sheep mass mortalities was collected in French and English medieval and Early Modern written sources, including historical texts (chronicles and annals), economic data (mostly from manorial records) and didactic texts (agricultural and veterinary treatises). All mentions of cause of death were noted, and where relevant, specific diseases were identified on the basis of their symptomatology and designation (see [81]). This allowed us to identify a list of the most common causes for mass ovine mortalities in different time periods; for the seventeenth to nineteenth centuries, disease-related causes included liver fluke, sheeppox, anthrax and Sologne red disease, and accidental causes included grain overload, hunger, hypothermia, wolf and dog predation, lightning strike and drowning [15]. Each of these causes of death was then described according to 16 parameters (historical presence, geography, terrain, seasonality, contagiousness, mortality rates, affected species, racial influence, age profile, sex profile, impact of physiological status, impact of sanitary status, bone lesions, carcass aspect, others), taking into account both historical data describing their past presentation and present-day veterinary knowledge. This information was summarized in 12 fact sheets available in [15].

The parameters obtained from the archaeological and osteological analysis of the Louvres ‘le Bouteiller’ deposit were then compiled into an epidemiological profile (electronic supplementary material, table S2) and compared to that of the 12 mortality causes, to evaluate fit and identify potential diagnostic hypotheses.

### 7.3 Ancient DNA laboratory work

All ancient DNA laboratory work was performed in dedicated ancient DNA facilities in Trinity College Dublin, Ireland. Personal protective equipment was used at all times, including boiler suits, hair nets, gloves (two layers), and face masks. All equipment and surfaces were regularly cleaned with dilute (0.5%) sodium hypochlorite. Polymerase chain reaction (PCR) amplification reactions were performed in different laboratory facilities to avoid contamination of amplified materials.

A total of five ovine petrosal bones, 10 teeth, and one dental calculus sample were processed. Petrosal bones were subsampled using a Dremel saw at the densest region. Bone fragments were then pulverized using a MixerMill at 30 Hz until fully pulverized. Teeth were sectioned along the cementoenamel junction, and each root was further halved. Material from the pulp chamber and inside the root was collected using a dental drill, yielding between 8 and 59 mg of powder per tooth. Dental calculus from sheep no. 2 was removed using a sterile blade, generating a total of 20 mg of material.

### 7.4 Bones, teeth and calculus extraction

Bone, tooth and calculus material were first subjected to a 15–30 min pre-digestion in ethylenediamine-tetraacetic acid (EDTA) (15 minutes only for the calculus sample). For teeth samples, both the EDTA wash and the remaining material pellet underwent a 24 h digestion in EDTA and proteinase K at 37°C, following the extraction steps in [82]. After incubation, DNA was purified using a modified pH-adjusted phosphate buffer (PB) binding buffer [82].

### 7.5 Nodule extraction protocol

The pulverized nodule was digested using a similar protocol as described in [82], but removing bleach pre-wash. Briefly, 1 ml 0.5 M EDTA was added to the nodule powder and also an empty tube (extraction blank). Tubes were incubated at 37°C for 30 min, with constant rotation. Tubes were briefly centrifuged to 13 300 rpm and the EDTA was removed and stored. An extraction buffer was prepared (40 μl 1M Tris-HCl, 34 μl sodium dodecyl sulfate (SDS), 1880 μl 0.5M EDTA, 26 μl proteinase K), subjecting the buffer to 30 min of UV prior to the addition of proteinase K. One millilitre of extraction buffer was then added to each tube and the tubes vortexed. Tubes were incubated for approximately 24 h at 37°C and gentle agitation.

After digestion, tubes were spun at 13 300 rpm for 10 min to separate supernatant and any remaining nodule. The supernatant was then subject to purification using large volume Roche columns and 13 μl of a modified PB buffer (added to 500 ml modified PB: 16 ml 3 M sodium acetate, 13.2 ml 5 M sodium chloride). After spin-down and purification, DNA was eluted in 50 μl elution buffer and tween (EBT).

### 7.6 Sequencing library building

All libraries were double stranded DNA libraries constructed following the Meyer & Kircher protocol [83]. One library for each sample was prepared without uracil DNA glycosylase treatment using 12–16 μl starting DNA, to capture native damage patterns. All other prepared libraries were pre-treated with 5 μl UDG-glycosylase (New England Biolabs, M5505L) at 37°C for 1 h. Following dual indexed-PCR amplification for 12 cycles, libraries were shotgun sequenced on an Illumina Novaseq X (150 bp pair end). Sequencing statistics and metadata for each indexed amplification are provided in the electronic supplementary material, table S3.

### 7.7 Sequencing data processing and pathogen detection

Raw sequencing reads were trimmed and quality filtered using AdapterRemoval v. 2.3.2 [84], discarding reads shorter than 30 bp and merging overlapping pairs (—collapse—minadapterover-lap 1—adapter1 AGATCGGAAGAGCACACGTCTGAACTCCAGTCAC—adapter2 AGATCGGAAGA GCGTCGTGTAGGGAAAGAGTGT—minlength 30—trimns—trimqualities). To remove host-derived sequences, libraries were aligned to a concatenated reference comprising human and multiple animal genomes (GRCh38, Sscrofa11.1, ARS-UCD1.2, mCerEla1.1, ASM170441v1, EquCab3.0, ARS-UI_Ramb_v2.0). Metagenomic profiles were generated from the resulting unmapped reads using KrakenUniq v1.0.4 [23] and a custom database built from a microbial version of the NCBI ‘nt combined’ dataset supplemented with human and complete eukaryotic reference genomes.

To evaluate the reliability of taxonomic assignments, we applied the scoring framework of Guellil *et al*. [85] to compute an *E*-value calculated at the species level to distinguish between true and false positives in KrakenUniq taxonomic assignment. Taxa were subsequently filtered for a predefined panel of animal and human pathogens (electronic supplementary material, table S5), retaining only assignments with *E*-value >0.001 and ≥20 supporting reads. To validate the *E*-score signal of SPPV in the Louvres ‘le Bouteiller’ samples, we ran a mapping-based detection with HOPS [47] on positive libraries using the approach and database described in [8].

### 7.8 RNA bait enrichment for sheeppox virus DNA

A set of custom in-solution 3×-tiled 80 bp RNA baits (myBaits®) was designed and synthesized by Daicel Arbour Bioscience, targeting *Capripoxvirus* DNA. The bait design was based on reference genomes sequences for *Capripoxvirus* members (SPPV: NC_004002.1; GTPV: NC_004003.1; LSDV, NC_003027.1), with low complexity regions masked. Baits matching to host livestock genomes (sheep: GCF_016772045.2; goat: GCF_001704415.2_ARS1.2; cattle: GCF_002263795.3_ARS-UCD2.0) were excluded (five baits), as were baits with more than 35% repeat content. The final set of 80 bp baits amounts to 15 646 unique probes, designed to enrich across *Capripoxvirus*.

### 7.9 *Capripoxvirus* capture application

Amplified, dual index-incorporated libraries were targeted for enrichment for SPPV DNA, through application of the RNA baits described above. Amplified, multiplexed libraries were combined into a pool. The pool was desiccated and worked back up to 7 μl of laboratory-grade H_2_O. The pool was then subject to overnight (approx. 20 h) capture using the RNA baits, following the manufacturer’s instructions and making recommended modifications for degraded DNA (‘high sensitivity’), including setting the annealing temperature to 55°C and performing a second round of annealing and amplification (eight cycles) after the first amplification (16 cycles). Amplification was performed using KAPA HiFI Hotstart (Roche). Purified, captured libraries were then subject to shotgun sequencing for 150 bp paired-end reads on NovaSeq X platforms (Macrogen Europe, Amsterdam).

### 7.10 Analysis of sheep DNA

To analyse host DNA, the collapsed reads were aligned on the sheep reference genome Rambouillet v2 (ARS-UI_Ramb_v2.0) using the bwa aln algorithm [86] with relaxed parameters (-l 1024 n 0.01 -o 2). Reads with mapping quality under 30 were filtered out with samtools v. 1.19.2 [87], and duplicates were removed with Picard MarkDuplicates v. 2.26.11 (https://github.com/broadinstitute/picard). We calculated post-mortem damage patterns on the *Ovis aries* alignment using mapDamage2 [88], which are shown in the electronic supplementary material, figure S3.

To determine the genetic sex of sheep individuals, we assessed the ratio of reads aligning to autosomal and X chromosome contigs versus contig length [89]. Individuals with two X chromosomes should show the same read number-contig length relationship among investigated chromosomes, while individuals only carrying one X chromosome (that is, genetic males) should show depressed read alignment for X chromosome contigs. We report the genetic sex of each individual in tables 1 and 2.

To assess allele sharing between sheep [21] genome alignments, we used ANGSD [21] v. 0.937-74-g9100f3d to calculate *D*-statistics among sheep alignments for the nodule (RF001) and four of the petrous bone specimens (RF002-RF005, corresponding to individuals 1, 2, 4 and 8, respectively). The following parameters were used: -doAbbababa 1 -doCounts 1 -minQ 20 -minMapQ 30 -rmTrans 1. The *D*-scores and bootstrapped standard error (s.e.) estimates for these comparisons are presented in the electronic supplementary material, table S4.

Consensus mtDNA sequences were called from petrous and nodule aligned data on the sheep mitochondrion (NC_001941.1) with angsd doFasta (-doCounts 1 -trim 4 -setMinDepth 3 -setMaxDepth 100 -minQ 20 -minMapQ 30). A multiple sequence alignment was then produced by aligning consensus sequences with NC_001941.1 by using MAFFT with default parameters. A maximum likelihood tree was built with IQ-Tree2 [24] employing the nucleotide substitution model HKY+F as determined by ModelFinder [90].

### 7.11 Analysis of *Taenia hydatigena* DNA

To validate the presence of *T. hydatigena* in the nodule DNA, we aligned host (sheep)-removed reads on the mitochondrial genome of the *T. hydatigena* parasite (NC_012896.1), using the same steps and parameters used for alignment to the sheep genome. A consensus mtDNA sequence from the resulting bam file was then produced by using angsd doFasta (-doCounts 1 -trim 4 -setMinDepth 3 -setMaxDepth 100 -minQ 20 -minMapQ 30). To validate the *T. hydatigena* identity of the nodule DNA, the consensus sequence was aligned using MAFFT [91] with *Taenia asiatica* (NC_004826.2), *Taenia crassiceps* (NC_002547.1), *Taenia ovis* (NC_021138.1), *Taenia pisiformis* (NC_013844.1), *Taenia saginata* (NC_009938.1), *Taenia solium* (NC_004022.1) and *T. hydatigena* (NC_012896.1) mitochondrial genome. A maximum likelihood phylogeny of *Taenia* diversity was built with IQ-Tree2 [24] employing the nucleotide substitution model TIM+F+I+G4 as determined by ModelFinder [90] and rooted on *T. crassiceps* (NC_002547.1).

After validating the phylogenetic placement of the Louvres ‘le Bouteiller’ nodule DNA as being *T. hydatigena*, we decided to test for phylogenetic placement of the nodule DNA into *T. hydatigena* modern mitochondrial diversity. The same process was applied as described below by using the full diversity of A and B *T. hydatigena* haplogroups (see figure 4B). The maximum likelihood was built using the nucleotide substitution model TN+F+R2 as determined by ModelFinder [90] and the tree was midpoint rooted.

### 7.12 Analysis of sheeppox virus DNA

After alignment on the host, unaligned UDG-treated and captured libraries were aligned to a modified SPPV reference genome (NC_004002.1) using the bwa aln algorithm with relaxed parameters (-l 1024 n 0.01 -o 2). The SPPV genome contains two identical ITR regions at its extremities, causing reads derived from these regions to map ambiguously to both ends and receive a mapping quality of zero, which would remove them from downstream analyses. To avoid this, we truncated the terminal 2,213 bp from the reference genome using seqkit [92], allowing unique alignment of reads to the remaining extremity. Alignments with a mapping quality <30 were discarded using samtools v. 1.19.2, and PCR duplicates were removed with Picard MarkDuplicates v. 2.26.11. BAM files originating from the same samples were subsequently merged using Picard MergeSamFiles v. 2.26.11.

Consensus sequences were generated by calling variants with FreeBayes v. 1.3.6 (https://github.com/freebayes/freebayes), using minimum coverage thresholds of 100× for individual 1, 6× for individual 2, and 1× for individual 9 and requiring ≥90% support for alternative allele calls (—report-monomorphic—min-alternate-count mincov—min-coverage mincov—min-mapping-quality 30—min-alternate-fraction 0.90—ploidy 1). Variant call files were then filtered with bcftools view to mark low-quality positions as missing. Final consensus sequences were produced with bcftools consensus, masking missing sites, low-coverage positions and low-quality variants as N (-a N -M N).

Consensus sequences were then aligned with 16 modern SPPV sequences (electronic supplementary material, table S6) and the GTPV reference sequence (NC_004003.1) with MAFFT [91] (—adjustdirection—maxiterate 1000). SNPs were then extracted from the resulting alignment with snp-sites v. 2.5.1 by extracting only ACGT positions. A maximum likelihood tree was then built with IQ-Tree2 [24] using the TVMe+ASC substitution model selected by ModelFinder [90] and 1000 ultrafast bootstrap replicates and was rooted on GTPV.

To check for nucleotide variation between the Louvres ‘le Bouteiller’ genomes, a SNP distance matrix was computed on the resulting set of SNP with snp-dists (https://github.com/tseemann/snp-dists).

## Supplementary Material

Supplementary material is available online at https://doi.org/10.6084/m9.figshare.c.8490923.

SI

SI Tables

## Figures and Tables

**Table 1:** Osteoarchaeological analysis of the sheep skeletons from feature 3, Louvres ‘le Bouteiller’.

individual ID	DNA ID	minimum elements	withers height (cm)	sex (osteological)	age at death (years)	cut marks	pathology
Ind. 1	RF002	72	63.5	male	4.5-8	no	minor
Ind. 2	RF003	94	54.9	female	>7	yes	yes— nodule
Ind. 3	n/a	75	59.4	female	>7	no	minor
Ind. 4	RF004	98	61.2	ambiguous	4.5–6	no	minor
Ind. 5	n/a	28	56.4	female	older adult	yes	no
Ind. 6	RF006	88	59.6	female	4.5–8	no	no
Ind. 7	n/a	38	61.9	male	>7	no	minor
Ind. 8	RF005	42	63.4	male	2.5–3.5	no	minor
Ind. 9	RF009	34	61.9	male	>7	no	no

**Table 2:** Details of Louvres ‘le Bouteiller’ samples analysed and associated host DNA statistics, including sample identification, material type, sampling location, endogenous DNA content, mitochondrial DNA (mtDNA) coverage, host species and genetically determined sex. (DNA damage levels are reported as cytosine-to-thymine misincorporation frequencies at the 5′ end (5pC→T) at the first base of sequencing reads (position 1).)

Individual ID	DNA ID	material	sample location	endogenous DNA (%)	mthNA coverage (×- fold)	5pCto T pos1	host Species	sex (DNA)
Ind. 1	RF002	bone, petrous	within centre of bone	46.71	58.72	0.06	*O. aries*	male
Ind. 2	RF003	bone, petrous	within centre of bone	59.5	68.78	0.12	*O. aries*	female
Ind. 4	RF004	bone, petrous	within centre of bone	39.64	14.64	0.16	*O. aries*	female
Ind. 8	RF005	bone, petrous	within centre of bone	48.37	61.64	0.12	*O. aries*	male
Ind. 9	RF009-P	bone, petrous	within centre of bone	16.09	3.53	0.24	*O. aries*	male
Ind. 1	RF002-M	tooth, lower M1, left	within pulpar cavity	0.05	0.06	0.33	*O. aries*	male
Ind. 4	RF004-M	tooth, lower M1, right	within pulpar cavity	1.04	0.43	0.13	*O. aries*	female
Ind. 8	RF005-MAN	tooth, lower M1, right	within pulpar cavity	0.02	0	0.22	*O. aries*	male
Ind. 1	RF002-M2L	tooth, lower M2, left	within pulpar cavity	0.94	0.2	0.14	*O. aries*	male
Ind. 2	RF003-M	tooth, lower M2, left	within pulpar cavity	0.1	0.56	0.05	*O. aries*	female
Ind. 8	RF005-M	tooth, lower M2, right	within pulpar cavity	0.25	1.31	0.00	*O. aries*	male
Ind. 9	RF009-MAX	tooth, upper M1, right	within pulpar cavity	0.75	0.23	0.26	*O. aries*	male
Ind. 4	RF004-MAX	tooth, upper M3, left	within pulpar cavity	0.25	0.11	0.14	*O. aries*	female
Ind.6/7	RF006-M3D	tooth, upper M3, right	within pulpar cavity	20.54	3.56	0.10	*O. aries*	female
Ind. 2	RF003-M-Cal	dental calculus	scraping from tooth buccal surface	0.8	n/a	n/a	*O. aries*	/
Ind. 2	RF001	pathological nodule	complete pulverized nodule	22.36	84.24	0.17	*O. aries*	female

**Table 3:** Samples selected for pathogen identification and results of DNA analysis. (For each sample, pathogen presence, final genome coverage, breadth of coverage at ≥1×, average nucleotide identity (ANI), and evidence of DNA damage are reported for sheeppox virus (SPPV) and *T. hydatigena*. ‘ins. reads’ indicates insufficient or absence of reads mapped for reliable analysis.)

individual ID	DNAID	material	pathogen detected	SPPV final genome coverage *(x-* fold)	SPPV breadth = 1×	SPPVANI	SPPV damage	*T. hydatigena* final genome coverage (*x-*fold)	*T. hydatigena* breadth = 1×	*T. hydatigena* ANI	mthNA *Taenia hydatigena* coverage	*Taenia hydatigena* damage
Ind. 1	RF002	petrous	n/a	n/a	~0	~0	ins. reads	n/a	n/a	n/a	n/a	ins. reads
Ind. 1	RF002-M	tooth	n/a	n/a	~0	~0	ins. reads	n/a	n/a	n/a	n/a	Ins. reads
Ind. 1	RF002-M2L	tooth	SPPV	12.1	99.9	99.5	yes	n/a	n/a	n/a	n/a	ins. reads
Ind. 2	RF003	petrous	n/a	n/a	~0	~0	ins. reads	n/a	n/a	n/a	n/a	ins. reads
Ind. 2	RF003-M	tooth	SPPV	263.0	100	99.6	yes	n/a	n/a	n/a	n/a	ins. reads
Ind. 4	RF004	petrous	n/a	n/a	n/a	n/a	ins. reads	n/a	n/a	n/a	n/a	ins. reads
Ind. 4	RF004-M	tooth	SPPV	~0	~0	~0	ins. reads	n/a	n/a	n/a	n/a	ins. reads
Ind. 4	RF004-MAX	tooth	n/a	n/a	n/a	n/a	ins. reads	n/a	n/a	n/a	n/a	ins. reads
Ind. 6/7	RF006-M3D	tooth	n/a	n/a	n/a	n/a	ins. reads	n/a	n/a	n/a	n/a	ins. reads
Ind. 8	RF005	petrous	n/a	n/a	n/a	n/a	ins. reads	n/a	n/a	n/a	n/a	ins. reads
Ind. 8	RF005-MAN	tooth	n/a	n/a	n/a	n/a	ins. reads	n/a	n/a	n/a	n/a	ins. reads
Ind. 8	RF005-M	tooth	n/a	n/a	n/a	n/a	ins. reads	n/a	n/a	n/a	n/a	ins. reads
Ind. 9	RF009-P	petrous	n/a	n/a	n/a	n/a	ins. reads	n/a	n/a	n/a	n/a	ins. reads
Ind. 9	RF009-MAX	tooth	SPPV	1.5	77.2	99.5	yes	n/a	n/a	n/a	n/a	ins. reads
Ind. 2	RF001	nodule	*T. hydatigena*	n/a	n/a	n/a	ins. reads	0.24	18.3	99.6	130.8	yes
Ind. 2	RF003-M-Cal	calculus	SPPV	~0	~0	~0	ins. reads	n/a	n/a	n/a	n/a	ins. reads

## Data accessibility

All raw sequencing data generated here is available on the European Nucleotide Archive, under Project Accession PRJEB103957.
